# Receptor tyrosine kinases: role in cancer progression

**DOI:** 10.3390/curroncol13050019

**Published:** 2006-10

**Authors:** V. Sangwan, M. Park

**Affiliations:** * Molecular Oncology Group, McGill University Health Centre, Montreal, Quebec; † Departments of Medicine, Biochemistry and Oncology, McGill University, Montreal, Quebec

**Keywords:** Receptor tyrosine kinase, cancer

## INTRODUCTION

Tight control of cell proliferation and morphogenesis in conjunction with programmed cell death (apoptosis) is required to ensure normal tissue patterning. Alterations that cause an imbalance of the cellular signals that control these events may promote cell growth, suppress apoptosis, and enhance cell invasion, resulting in oncogenesis.

Many of these signals are regulated by receptor tyrosine kinases (rtks). These rtks are membrane-spanning proteins with intrinsic phosphotyrosine kinase activity. Their activity is normally tightly regulated. In the absence of ligand, rtks reside in the plasma membrane as inactive enzymes. Binding of ligand promotes receptor dimerization and a change in conformation that leads to activation of the kinase and transphosphorylation of the receptor on specific tyrosine residues.

Phosphorylated tyrosine residues provide docking sites on the receptor for signalling proteins that recognize phosphotyrosine residues and that act to relay the signal from the receptor at the plasma membrane into the cell 1. Once activated, a normal cell has mechanisms in place to downregulate the receptor through receptor internalization. Endocytosis and eventual lysosomal degradation and ubiquitination of rtks has been shown to play a key role in these processes 2; however, when altered through mutation or chromosomal translocation, or after genomic amplification, many rtks can become potent oncogenes by delivering a continuous or enhanced signal.

In addition to alterations in the rtk, aberrant degradation of rtks is associated with numerous cancers, underscoring the importance of this mechanism of regulation of rtks 3,4. To date, 58 genes encoding rtks have been identified in the human genome, 30 of which have been found to be dysregulated in human cancers 5.

## DISRUPTED SIGNALLING BALANCE

Many cells *in vivo* are in a quiescent state (G0) with unduplicated dna. The balance between growth inhibitory signals—provided by soluble factors such as transforming growth factor beta (tgfb), cell-to-cell contacts, and adhesion to extracellular matrix components —and growth stimulatory signals (through growth-factor activation of rtks) forces cells to enter G1 and initiate cell division 6. Successful transition through the G1 phase requires sustained growth factor stimulation over a period of several hours, followed by the presence of a progression factor such as insulin-like growth factor 1 (igf-1).

This dual signal requirement may prevent accidental triggering of quiescent cells into the cell cycle by transient exposure to mitogenic growth factors. In some cell types, the absence of growth-factor stimulation causes the rapid onset of programmed cell death (apoptosis). Therefore, non-scheduled expression of a growth factor may result in a constant stimulation of cell growth in addition to a block in apoptosis.

A variety of tumour cells are known to produce and release growth factors for which these cells bear receptors and can respond. For example, lung cancers produce several growth factors—tgfb , stem cell factor, and igf-1—as well as the receptors for these ligands [epidermal growth factor receptor (egfr), Kit R, and igf-1R respectively] 7. Melanomas express basic fibroblast growth factor (bfgf), which they require for proliferation, and carcinomas that express high levels of the receptors egfr and Met secrete the corresponding factors tgfb or hepatocyte growth factor (hgf). These signalling loops appear to be functionally important for tumour growth and are associated with short patient survival times.

In addition to having a direct function in tumour cells, growth factors and rtks play key roles in modulating the tumour microenvironment. Most carcinomas depend on their microenvironment for survival through the recruitment of fibroblasts, macrophages, and vasculature. For example, carcinoma cells frequently release platelet-derived growth factor (pdgf), which acts to recruit and activate stromal cells that express the pdgf receptor. Those cells in turn release mitogenic igfs and epithelial invasion and motility factors such as hgf.

The construction of the vasculature is also regulated through multiple interactions between growth factors and rtks, including pdgf, bfgf, and vascular endothelial growth factor 8. Similarly, macrophages recruited to the tumour microenvironment through the production of colony stimulating factor-1 (csf-1) by breast carcinomas can themselves release growth factors such as egf that can enhance both the proliferation of and invasion by tumour cells 9.

## RATIONALE FOR TREATMENT

The identification, in multiple cancers, of alterations in rtks and of dysregulated rtk signalling has provided the rationale for anti-rtk drug treatment. Trastuzumab, imatinib, and gefitinib are some of the first examples of drugs that have successfully translated basic research on rtks and oncogenes into cancer therapeutics 10. Strategies aimed at preventing and inhibiting rtk signalling include antibodies to the extracellular domain of the receptor (such as trastuzumab), antagonist ligands, small-molecule inhibitors of protein tyrosine kinase activity (imatinib and gefitinib), and peptide mimetics that act as inhibitors of protein–protein interactions.

## CLINICAL STUDIES

Since the mid-1990s, clinical studies have established that tyrosine kinase inhibitors are relatively safe and therapeutically active in target patients whose tumour growth is driven by tyrosine kinases. Trastuzumab is a humanized antibody that recognises the extracellular domain of the her2 rtk, which is amplified in approximately 25% of invasive primary breast cancers.

The family of rtks to which her2 belongs also includes egfr (her1), her3, and her4. No ligand has been identified for her2 (as has occurred with its family members); instead, her2 forms heterodimers with other family members (her1–4), enhancing and diversifying their signalling in response to ligand stimulation. Binding of trastuzumab to the extracellular domain of her2 enhances downregulation of her2 (including its binding partners) from the cell surface, decreasing cellular signalling. Trastuzumab was shown to offer clinical benefit to patients with her2-positive metastatic breast cancer, and recent trials have demonstrated highly significant survival benefits to her2-positive patients when trastuzumab is used in combination with chemotherapy 11,12. Similar strategies have been developed for egfr, where the anti-egfr monoclonal antibody cetuximab has clinical benefit when combined with radiotherapy for head-and-neck cancers.

In addition to monoclonal antibodies, small-molecule inhibitors that block the catalytic activity of rtks have demonstrated success in the clinical setting. The small-molecule inhibitors for egfr (gefitinib and erlotinib) show modest antitumour activity when administered as single agents in unselected patient populations with non-small-cell lung carcinoma, but they show a positive clinical response in a subset of patients that harbour somatic mutations in the egfr gene and whose tumours may be dependent on expression of those mutated genes 13.

Imatinib, originally designed as an inhibitor of the non-rtk tyrosine kinase bcr-abl and found in patients with chronic myelogenous leukemia (cml) with chromosome 9–22 translocations, has shown significant therapeutic success in bcr-abl–positive cml and acute lymphoblastic leukemia. However, in addition to its activity for bcr-abl, imatinib also blocks the activity of several rtks, including the Kit and pdgf receptors, and has shown robust clinical activity in a subset of gastrointestinal stromal tumours and in chronic myelomonocytic leukemia harbouring mutations in the Kit or pdgf receptors 14.

As demonstrated for other anticancer agents, patients acquire resistance after prolonged treatment, and surprisingly, much of this resistance stems from secondary mutations within the target rtk and mutations that involve downstream pathways activated by rtks. Combination-type therapies that target multiple pathways regulated by rtks will increase efficacy and may reduce resistance.

## SUMMARY

The mutation and deregulation of rtks can lead to a wide variety of changes in cellular structure and function, all of which contribute to the malignant phenotype and pathologic behaviour of human cancer. The progress in recent years on the clinical validation of molecularly targeted drugs that inhibit rtks has demonstrated that, in appropriately selected patients, treatment can alter disease progression and survival.

Rational drug design will benefit from computational biology and a detailed understanding at the structural level both of the kinase domains and of the ligand binding sites of these receptors. The large tissue and serum repositories from patients in clinical trials will also allow outcome to be linked to molecular changes observed in tumours.

## References

[1] Pawson T, Scott JD (2005). Protein phosphorylation in signalling— 50 years and counting. Trends Biochem Sci.

[2] Wiley HS, Burke PM (2001). Regulation of receptor tyrosine kinase signaling by endocytic trafficking. Traffic.

[3] Peschard P, Park M (2003). Escape from *cbl-*mediated downregulation: a recurrent theme for oncogenic deregulation of receptor tyrosine kinases. Cancer Cell.

[4] Bache KG, Slagsvold T, Stenmark H (2004). Defective downregulation of receptor tyrosine kinases in cancer. Embo J.

[5] Blume–Jensen P, Hunter T (2001). Oncogenic kinase signalling. Nature.

[6] Jones SM, Kazlauskas A (2001). Growth-factor-dependent mitogenesis requires two distinct phases of signalling. Nat Cell Biol.

[7] Maulik G, Kijima T, Salgia R (2003). Role of receptor tyrosine kinases in lung cancer. Methods Mol Med.

[8] Folkman J (2000). Incipient angiogenesis. J Natl Cancer Inst.

[9] Condeelis J, Pollard JW (2006). Macrophages: obligate partners for tumor cell migration, invasion, and metastasis. Cell.

[10] Baselga J (2006). Targeting tyrosine kinases in cancer: the second wave. Science.

[11] Romond EH, Perez EA, Bryant J (2005). Trastuzumab plus adjuvant chemotherapy for operable her2-positive breast cancer. N Engl J Med.

[12] Piccart–Gebhart MJ, Procter M, Leyland–Jones B (2005). Trastuzumab after adjuvant chemotherapy in her2-positive breast cancer. N Engl J Med.

[13] Paez JG, Janne PA, Lee JC (2004). *EGFR* mutations in lung cancer: correlation with clinical response to gefitinib therapy. Science.

[14] Heinrich MC, Corless CL, Demetri GD (2003). Kinase mutations and imatinib response in patients with metastatic gastrointestinal stromal tumor. J Clin Oncol.

